# Effects of *Gryllus bimaculatus* Powder on Physicochemical Properties and Consumer Acceptability of 3D-Printed Gluten-Free Chocolate Cookies Using Survival Analysis

**DOI:** 10.3390/foods14132291

**Published:** 2025-06-27

**Authors:** Woonseo Baik, Dongju Lee, Youngseung Lee

**Affiliations:** Department of Food Science and Nutrition, Dankook University, Cheonan-si 31116, Republic of Korea; dnstjsms@naver.com (W.B.); dongju042027@naver.com (D.L.)

**Keywords:** edible insect, gluten free, cookie, survival analysis

## Abstract

To mitigate consumer aversion toward edible insects, it is essential to determine the optimal level of insect powder by considering consumer acceptability. In this study, gluten-free (GF) chocolate cookies were manufactured using 3D printing with varying concentrations (0, 3, 6, 9, 12, and 15%) of *Gryllus bimaculatus* (GB) powder. Physicochemical properties, sensory perception using rate-all-that-apply questions, and consumer acceptability using survival analysis were evaluated. The effects of GB powder concentration on the proximate composition, pH, color attributes, physical properties, 3D printing performance, and post-processing of the cookies were analyzed and discussed. As the concentration of GB powder increased, crude protein, ash, crude fat, a*, and mechanical force increased, while L*, b*, and the pH of both the dough and cookies decreased. Consumer tests showed a negative correlation between GB concentration and consumer acceptability, with cookies containing 3% GB receiving the highest overall liking scores. Principal component analysis and partial least squares regression showed that lower GB levels enhanced positive sensory attributes such as sweetness, chocolate flavor, and moistness, whereas higher levels intensified bitter taste and astringency, contributing to reduced acceptability. According to survival analysis, the GB concentration at which 50% of consumers were predicted to reject the product was estimated at 5.23%, indicating the necessity to limit GB incorporation below this threshold to ensure consumer acceptance. This study provides a comprehensive understanding of the quality characteristics and consumer acceptability of insect-based GF cookies, offering valuable insights for future product development and market applications.

## 1. Introduction

Greenhouse gas emissions resulting from anthropogenic activities have accelerated global warming, posing a critical threat to global food production and food security [1]. Among the major contributors to greenhouse gas emissions is the livestock sector, necessitating the development of alternative strategies to reduce meat consumption and alleviate reliance on conventional animal agriculture [2]. In this context, increasing attention has been directed toward sustainable alternative protein sources. Among these, edible insects, which have long been consumed in various cultural contexts, have recently gained significant global recognition as a viable and environmentally friendly protein option [3]. Edible insects are characterized by high reproductive efficiency, rapid growth rates, excellent feed conversion, and minimal environmental impact [4]. They also require significantly less space and fewer resources than traditional livestock, further enhancing their economic viability and underscoring their potential as a sustainable protein source [3].

Among various insect species, *Gryllus bimaculatus* (GB) is one of the most widely consumed edible insects globally, owing to its high nutritional value [4]. According to a compositional analysis of five commercially available edible insect species conducted in the Republic of Korea, GB contains approximately 58.3% crude protein, 11.9% fat, 9.5% crude fiber, 9.7% ash, and 10.6% nitrogen-free extract on a dry matter basis [5]. It is also rich in essential amino acids such as methionine, lysine, histidine, valine, and leucine. In particular, the fat content of GB includes health-beneficial fatty acids such as linoleic acid and stearic acid. Notably, stearic acid, despite being a saturated fatty acid, has been reported to have a neutral or even favorable effect on blood cholesterol levels [6]. Additionally, chitin, a primary insoluble fiber found in crickets, has been reported to support digestive health and function as a prebiotic by stimulating the growth of beneficial gut microbiota [4]. However, despite the nutritional and environmental advantages of edible insects, consumers often exhibit disgust and fear toward their consumption, leading to general avoidance [7]. To address these negative perceptions, strategies such as processing insects into unrecognizable forms or incorporating them into familiar food products have been actively proposed [7].

Concurrently, the prevalence of gluten-related disorders, including celiac disease, non-celiac gluten sensitivity, wheat allergy, and irritable bowel syndrome, has led to increased consumer demand for gluten-free (GF) products [8]. Strict adherence to a GF diet is essential for managing these conditions, and in recent years, GF foods have also gained popularity among health-conscious consumers regardless of medical necessity [9]. Despite the growing GF market, most commercial GF products rely heavily on carbohydrate-based ingredients such as corn, rice, and starch, resulting in low levels of protein, dietary fiber, and minerals and thereby presenting nutritional limitations [10]. To address these imbalances, the incorporation of functional ingredients with enhanced nutritional profiles has become imperative. Edible insects, with their high protein and fat content, offer promising potential to enhance the nutritional value of GF foods and serve as alternative protein sources [9].

Three-dimensional (3D) printing is an advanced manufacturing technology that fabricates complex structures by depositing successive layers of material based on a digital 3D model [11]. Owing to its versatility and precision, 3D printing has found applications across various industries including military, automotive, and textiles and is increasingly being explored in the food sector [12]. In particular, 3D food printing enables the modification of ingredient shape and texture, thus facilitating the development of novel sensory experiences and promoting the consumption of alternative protein sources such as edible insects [13]. Furthermore, additive manufacturing enables the production of customized food products tailored to individual nutritional needs and preferences, thereby contributing to health-oriented dietary solutions [14,15].

The use of food ink allows for precise control over ingredient composition, making it a viable method for formulating functional foods for individuals with dietary restrictions such as lactose intolerance or gluten sensitivity. However, the production of such foods requires not only optimized 3D printing processes but also appropriate post-processing techniques [16,17]. Post-processing methods are generally categorized into conventional cooking (e.g., baking, steaming, frying, etc.), advanced thermal techniques (e.g., laser cooking, infrared cooking, etc.), and dehydration methods [18]. Ensuring compatibility between 3D printing and traditional cooking methods is crucial for the scalability and industrial implementation of 3D food printing [18]. Thus, it is necessary to investigate the compatibility of printed food with conventional cooking processes and to assess how post-processing affects the final product quality.

One of the key challenges in the food industry is improving the consumer acceptability of insect-based foods [19]. Edible insects exhibit diverse sensory attributes, including appearance, aroma, flavor, texture, and even sound during mastication, with over 2000 species reported globally as potential food sources [20]. However, not all sensory attributes are perceived positively by consumers, particularly those unfamiliar with entomophagy, who may exhibit food neophobia and feelings of disgust, ultimately reducing their willingness to consume insect-based products [21]. Although altering the physical appearance of insect-based foods may partially mitigate consumer aversion, visual transformation alone is insufficient to overcome negative perceptions [19]. In addition, Ghosh et al. [22] emphasized that, beyond sensory attributes, various factors such as cultural background, prior experience with insect consumption, individual attitudes, and the visibility of insect components significantly influence consumer acceptance. Therefore, a comprehensive understanding of how consumers perceive and evaluate the sensory attributes of insect-based foods is essential for enhancing their acceptability. Consumer-based sensory evaluations play a significant role in improving familiarity and preference, enabling the development of products that align with consumer expectations [23].

Several studies have investigated the application of 3D printing using edible insects. These include evaluations of the printability of commercial insect gels and the role of protein and chitin in printability [2], studies on the 4D printing of edible insect-based materials with shape transformation upon thermal dehydration [13], the development of health snacks using *Tenebrio molitor* and *Alphitobius diaperinus* powders with 3D printing applications [24], and the use of insect protein as emulsifiers to produce high internal phase emulsions as fat substitutes for 3D printing [25].

Product quality is a critical determinant of consumer purchasing behavior. With the growing demand for fresh, safe, and high-quality food products, the food industry is increasingly required to define acceptable sensory defect thresholds and to accurately predict sensory shelf life using scientific methods [26]. In food science, survival analysis has traditionally been employed to estimate shelf life, treating storage time as the event-related variable. Subsequently, some studies have applied survival models using the concentration of specific components instead of time [27]. More recently, multivariate techniques like principal component analysis (PCA) have been used to derive quality deterioration indices, which are then modeled in relation to consumer rejection data using survival analysis to estimate sensory rejection thresholds or sensory shelf life [28]. This consumer-centric approach enables the establishment of failure criteria grounded in sensory perception, rather than relying on arbitrary or producer-defined limits, thereby improving the relevance and accuracy of product acceptability evaluations.

Although research on 3D printing applications for edible insects is progressing, there remains a lack of studies exploring the use of GB as a food ink component and its effects on the quality and consumer acceptance of 3D-printed cookies. Therefore, this study aims to provide valuable insights to support the edible insect industry, develop cookie dough formulations incorporating insect powder, and assess the physicochemical properties and consumer acceptability of 3D-printed cookies using survival analysis.

## 2. Materials and Methods

### 2.1. Materials

The following ingredients were used in this study: gluten-free wheat starch (Wecanfood, Seoul, Republic of Korea), refined sugar (CJ CheilJedang, Seoul, Republic of Korea), unsalted butter (WestGold, Emart, Seoul, Republic of Korea), cocoa powder (Mix & Bake, Samyang, Seongnam, Republic of Korea), vanilla extract (Low&Oil, Venta, Seoul, Republic of Korea), and refined salt (Daesang, Seoul, Republic of Korea). The wheat starch used in this study was labeled as gluten-free according to the distribution information provided by the manufacturer and is considered suitable for consumers sensitive to gluten. All ingredients, except eggs, were purchased through an online retailer (Emart-mall), while fresh eggs were obtained from a local supermarket in Cheonan, Korea. GB specimens were purchased in a mid-infrared dried form from Cricket Farm (Hwaseong, Republic of Korea) via an online order. The dried crickets were first coarsely ground using a mortar and pestle (Samhwa, Seoul, Republic of Korea), followed by fine grinding using a commercial blender (HR2096, Philips, Amsterdam, The Netherlands). The resulting powder was passed through a 60-mesh sieve (203–250 μm, Chunggye, Seoul, Republic of Korea) to ensure uniform particle size. The processed cricket powder was portioned into 100 g units, vacuum-sealed, and stored at −25 °C. All samples were used within two months of preparation for experimental consistency.

### 2.2. Cookie Dough Preparation

Chocolate cookie doughs were prepared according to the formulation presented in Table 1. A preliminary experiment showed that when GB powder was added at concentrations below 3%, the sensory differences between samples were minimal due to the dominant chocolate flavor, which likely masked the perceptible characteristics of the insect powder. Therefore, the experimental groups consisted of doughs in which 3, 6, 9, 12, and 15% (*w*/*w*) of wheat starch was replaced with GB powder (designated as CP3, CP6, CP9, CP12, and CP15, respectively), while the control group contained no insect powder. Initially, unsalted butter was softened at room temperature (25 °C) for 1 h and manually creamed for 5 min. Granulated sugar was then added in three portions and thoroughly mixed to achieve homogeneity. Subsequently, room-temperature eggs, salt, and vanilla extract were incorporated and blended for an additional 3 min. The wheat starch and GB powder were pre-sifted and added to the mixture, followed by 5 min of kneading to form the dough. The completed dough was rested under refrigeration at 4 °C for 30 min prior to further processing.

### 2.3. Three-Dimensional Printing and Post-Processing

**Figure 1:**
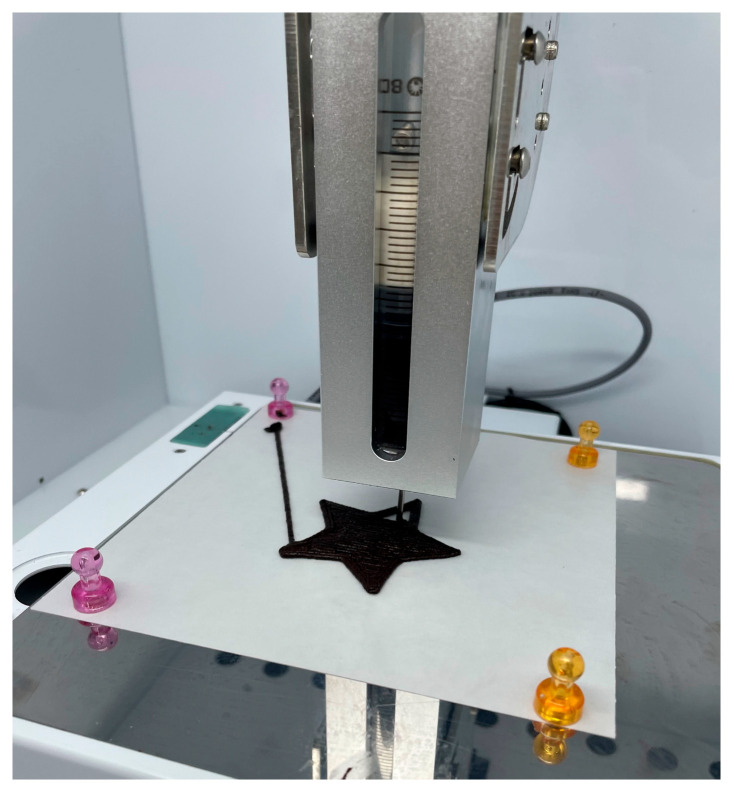
Three-dimensional printing of gluten-free chocolate cookie dough with star-shaped design using syringe-based extrusion system.

The cookie dough was stabilized at room temperature (25 °C) for approximately 30 min prior to loading into 60 mL cartridges (Becton-Dickinson Inc., Franklin Lakes, NJ, USA). The filled cartridges were then installed in a 3D food printer (YOLI LAB 1.0, YOLILO Co., Seoul, Republic of Korea) for printing (Figure 1). Before printing, slicing parameters were set using Cura software (version 2.4, Ultimaker BV, Federales, The Netherlands) with an infill density of 80%, a reticular infill pattern, and a printing angle of 90°. Preliminary trials involving various shapes revealed that a star-shaped design with a height of 2 mm exhibited superior structural stability during printing, with minimal layer separation or collapse. Additionally, this geometry effectively distributed mechanical stress through multidirectional protrusions and facilitated uniform heat transfer during baking due to its large surface area, thereby contributing to consistent moisture evaporation and product quality. These observations were consistent with previous findings indicating that shape and internal architecture significantly influence printability and post-processing stability in 3D-printed foods [15].

The printing conditions were set as follows: a nozzle diameter of 1.5 mm, first layer height of 1.8 mm, subsequent layer height of 1.7 mm, printing speed of 30 mm/s, and infill speed of 20 mm/s. These parameters fall within the effective range reported by Barrios-Rodríguez et al. [29] for semi-solid food materials using rice protein-based inks. Each cookie was printed in approximately 1 min and 43 s. Following printing, the cookies were baked in a convection oven (SK Magic EON-B420M, SK Magic Co., Seoul, Republic of Korea) at 120 °C for 30 min. After baking, the samples were cooled at room temperature and stored under refrigeration (4 °C) for three days prior to analysis.

### 2.4. Proximate Composition of Cookies

The proximate composition of chocolate cookies containing GB powder was analyzed in accordance with the standard methods of the Association of Official Analytical Chemists [30]. Moisture content was determined using the 105 °C hot air oven drying method; crude protein content was measured by the semi-micro-Kjeldahl method; crude fat content was determined using Soxhlet extraction; and ash content was assessed via the dry ashing method. All analyses were performed in triplicate, and the results are presented as the mean ± standard deviation.

### 2.5. pH and Density of Cookie Dough

The pH of chocolate cookie doughs formulated with varying levels of GB powder was measured by homogenizing 3 g of dough with 20 mL of distilled water using a magnetic stirrer (MS-H280-Pro, DLAB, Seoul, Republic of Korea) for 20 min. The mixture was then filtered, and the supernatant was analyzed using a calibrated pH meter (HI98163, Hanna Instruments, Seoul, Republic of Korea), following the method of Koh et al. [31].

Dough density was determined by adding 5 g of dough to a 50 mL graduated cylinder containing 40 mL of distilled water and recording the change in volume. The density was calculated as the weight-to-volume ratio (g/mL), in accordance with the method described by Koh et al. [31]. All measurements were conducted in quintuplicate, and mean values were used for analysis.

### 2.6. pH and Color Analysis of Cookies 

The pH of chocolate cookies containing GB powder was determined by homogenizing 3 g of cookie samples with 27 mL of distilled water through stirring for 30 min. The homogenate was filtered, and the supernatant was analyzed using a calibrated pH meter (HI98163, Hanna Instruments, Seoul, Republic of Korea). Measurements were performed five times per sample, and mean values were used for the analysis [31].

Color measurements were conducted using a colorimeter (RM200QC, Lovibond, UK) after calibration with a standard white plate (L* = 97.4; a* = 0.5; b* = 1.3). The color parameters were recorded according to the CIE (Commission Internationale de l’Éclairage) color space, specifically lightness (L*), redness (a*), and yellowness (b*). Each sample was measured in triplicate, and the average values were reported.

### 2.7. Physical Properties of Cookies

The cookie spread ratio was determined according to the AACC standard method 10-50D [32]. The diameter was measured by aligning six cookies horizontally, recording the total length, rotating them 90°, and re-measuring. The average of the two measurements was used. The height was determined by stacking six cookies vertically, measuring the total height, and then repeating the measurement after rearranging the stacking order. The spread ratio was calculated as the ratio of diameter to thickness (D/H) using Equation (1). All measurements were performed in triplicate, and the mean values were used for analysis [33].

(1) $$Spread\;ratio=\frac{Diameter}{Thickness}$$

The loss rate was calculated based on the weight of cookies before and after baking, comparing the changes between the control and experimental groups. This was computed using Equation (2) [31]. Cookie weights were measured precisely using an analytical balance (EPG214DC, Ohaus Corp., Parsippany, NJ, USA). All measurements were conducted five times per sample, and mean values were reported.

(2) $$Loss\;rate\;(\%)=\frac{Weight\;before\;baking-Weight\;after\;baking}{Weight\;before\;baking}\times 100$$

### 2.8. Textural Analysis of Cookies

**Figure 2:**
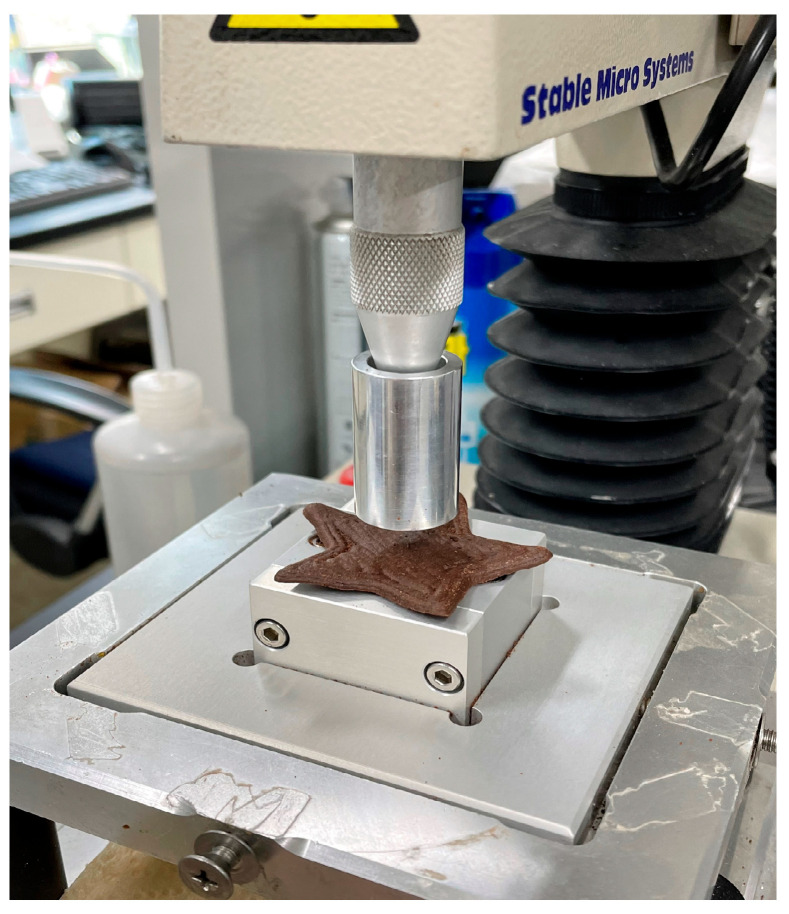
Single compression test of baked gluten-free chocolate cookies to evaluate mechanical force and energy.

The mechanical properties of chocolate cookies formulated with GB powder were evaluated using a texture analyzer (TA.XTplus, Stable Micro Systems, Godalming, UK) as shown in Figure 2. A cylindrical probe (P/20, diameter 20 mm) was employed under the following test conditions: trigger force 0.049 N, distance 5 mm, pre-test speed 1 mm/s, test speed 1 mm/s, and post-test speed 3 mm/s. All measurements were conducted at room temperature (25 °C). Hardness was assessed based on the maximum compression force (peak force, N) and the total area under the force–time curve. The peak force represents the maximum force required to fracture the cookie, while the total area reflects the overall distribution of force exerted during compression [34]. Each sample was measured 15 times, and mean values were used for analysis.

### 2.9. Consumer Test of Cookies

The consumer acceptance test was conducted following approval from the Institutional Review Board of Dankook University (Approval No.: DKU 2022-02-001), with participation from 100 subjects (81 females and 19 males). Prior to evaluation, cookie samples were equilibrated at room temperature (25 °C) for 1 h and individually presented to each panelist. Each sample was labeled with a random three-digit code, and the order of presentation was randomized using a Williams Latin Square design to minimize order bias. Participants were instructed to rinse their mouths with water between samples to avoid carryover effects.

Consumers were asked to assess their overall liking using a 9-point verbal category scale (1 = dislike extremely to 9 = like extremely). Consumers were asked to check all the terms that they considered to be appropriate to describe the samples and then rated the intensity of the relevant terms using a 5-point intensity scale (1 = “low intensity”; 3 = “medium”; and 5 =“high intensity”), which was referred to rate-all-that-apply (RATA) [35]. The presentation of the samples in the questionnaire was balanced within and across subjects using a Williams design to avoid presentation bias. Consumers were then asked the following question: “Would you normally consume this product? Yes or No?” This question aimed to determine whether the consumers would accept the product if the product was purchased or served. A survival analysis was performed to estimate the maximum acceptable concentration of GB powder that would not elicit consumer rejection, following the method described by Meneses et al. [36].

In addition, sensory attributes were categorized into four descriptors: appearance (brownness, fissureness, and density), aroma (butter aroma, vanilla aroma, caramel aroma, and chocolate aroma), taste (sweetness, saltiness, bitter taste, savory taste, chocolate taste, vanilla taste, oily taste, nutty taste, and ripe barley taste), and texture or mouthfeel (hardness, fracturability, cohesiveness, crispiness, moisture release, residual characteristic, cotton mouth, and loose particles).

### 2.10. Statistical Analysis

All statistical analyses were performed using XLSTAT software (version 2016 for Windows; Addinsoft, Inc., Paris, France). A one-way analysis of variance (ANOVA) was conducted to examine the statistical significance of differences among samples. When significant differences were detected (*p* < 0.05), Tukey’s post hoc test was applied to determine specific pairwise differences among sample means.

To investigate sensory attribute differences among samples based on consumer test data, PCA was conducted. Furthermore, partial least squares regression (PLSR) was employed to identify key sensory drivers influencing consumer liking by correlating overall acceptability scores with RATA attribute intensities. To predict consumer acceptance thresholds for the samples, survival analysis was conducted using MINITAB^®^ 16 (Minitab Inc., San Diego, CA, USA). Since consumers were exposed to predetermined concentration levels, the exact rejection concentration could not be directly observed in all cases, and thus, censoring was considered in the analysis. Specifically, consumers who rejected the lowest concentration level were classified as left-censored, while those who accepted lower concentrations but rejected higher concentrations were considered interval-censored. Participants who accepted all concentrations, including the maximum level provided, were classified as right-censored. Since the distribution of consumer rejection thresholds was not normally distributed, the exponential distribution was selected for survival data modeling.

## 3. Results and Discussion

### 3.1. Proximate Composition of Cookies

The proximate composition of chocolate cookies formulated with varying concentrations (3~15%) of GB powder is presented in Table 2. No significant differences were observed in moisture content among the samples, suggesting that the incorporation of cricket powder did not substantially alter the water retention capacity of the dough. This finding is consistent with a previous study on shortcake biscuits containing cricket powder, which also reported no significant variation in moisture content [37]. Such results indirectly imply that cricket powder, when used as a partial flour substitute in baked goods, may not adversely affect water-binding properties, thereby supporting its potential as a functional replacement ingredient.

In contrast, significant differences were observed in crude protein, crude fat, and ash contents among the samples (*p* < 0.05). Crude protein content increased significantly with higher levels of GB powder. The control group exhibited the lowest protein content (15.00%), while the CP3 sample showed a marked increase to 26.28%, which is approximately 1.75 times higher than that of the control. Protein content further increased in CP6 (28.98%), CP9 (31.10%), CP12 (32.76%), and CP15 (36.31%), indicating a strong positive correlation between insect powder addition and protein enrichment. This trend is attributed not only to the high protein content of cricket powder but also to the presence of nitrogen-containing polysaccharides such as chitin, glucosamine, and N-acetylglucosamine found in the insect exoskeleton, which may contribute to the overall protein-like content [10].

Crude fat content was the lowest in the control (15.84%) and ranged from 17 to 24% in samples containing cricket powder. According to Montowska et al. [38], commercial cricket powder typically contains between 23.6 and 29.1% crude fat, which aligns with the present findings. This is consistent with the known lipid profile of crickets, which includes high levels of palmitic acid (C16:0), oleic acid (C18:1), and linoleic acid (C18:2) [37]. Insect-derived lipids not only offer nutritional benefits but also influence dough structure and the generation of volatile compounds during baking, suggesting that cricket lipids significantly contributed to the observed changes in dough composition [10].

Ash content also showed a significant increase with increasing levels of cricket powder. The control had the lowest ash content (1.60%), whereas CP15 exhibited the highest value (2.05%). Since whole crickets were dried and powdered in this study, it is plausible that the high concentrations of calcium, phosphorus, and magnesium in the exoskeleton contributed substantially to the observed increase in ash content [39]. Indeed, ash content in insect powders has been reported to range between 2.95 and 5.22%, depending on the developmental stage and species, highlighting the structural contribution of mineralized body parts [39].

These results suggest that the addition of GB powder could serve to compensate for the nutritional deficiencies commonly found in GF products. Such products are typically based on starch-rich ingredients like corn or rice, which are often deficient in essential nutrients such as vitamins and minerals [10]. Cricket powder may thus be regarded as a functional food ingredient capable of enhancing the nutritional density of GF formulations—not only as a protein fortifier but also as a source of bioavailable minerals. However, given that insect-enriched cookies may differ from conventional products in sensory characteristics, an assessment of consumer acceptance and palatability is essential for quality optimization and successful product development.

### 3.2. pH and Density of Cookie Dough

The measurements of pH and density for chocolate cookie doughs containing various concentrations of GB powder are summarized in Table 3. The control exhibited the highest pH value (7.24), and a statistically significant decrease in pH was observed with increasing levels of cricket powder (*p* < 0.05). All experimental groups differed significantly from one another, indicating that the incorporation of cricket powder contributed to increased acidity in the dough. This reduction in pH is likely attributed to the intrinsic pH and chemical composition of the GB powder. Dried cricket powder typically exhibits a pH of approximately 6.3 [40], which has been associated with its high content of acidic amino acids such as glutamic acid and aspartic acid [41]. Additionally, organic acids and phenolic compounds generated during dry heat processing may be adsorbed onto the surface of the cricket powder and subsequently incorporated into the dough, thereby contributing to the observed decrease in pH [42]. Moreover, palmitic acid—comprising approximately 25% of the total fatty acids in GB—is a carboxylic acid that, upon thermal degradation, may yield low-molecular-weight organic acids such as acetic acid, further lowering the pH of the final product [43]. Collectively, these factors likely acted in combination to reduce the pH of the dough with increasing insect powder content.

In contrast, no significant differences were observed in dough density between the control and experimental groups, and no consistent trend was identified with increasing cricket powder levels. Dough density is a critical physical parameter in assessing product quality, as higher densities are generally associated with compact structures and increased hardness, whereas lower densities can result in greater porosity and more fragile textures [44]. Several factors may influence dough density, including the type of flour, water absorption capacity, lipid characteristics, and mixing methods and durations, all of which can affect the flavor and color of the final product [45]. Excessively high dough density may negatively impact consumer acceptability due to increased hardness, while overly low density may enhance spreadability and compromise structural integrity and visual appeal [46]. In light of these considerations, the results suggest that the addition of GB powder within the tested range did not markedly affect the density of the dough, implying that the use of edible insect powder may be feasible without compromising the structural stability of GF baked goods.

### 3.3. pH and Color Analysis of Cookies

The pH and color characteristics of chocolate cookies formulated with varying levels of GB powder are presented in Table 4. The pH of the cookies followed a trend similar to that observed in the dough, with the control exhibiting the highest value (6.97). In contrast, experimental samples containing insect powder showed a significant decrease in pH as the concentration of GB powder increased, indicating that the addition of cricket powder contributed to greater acidity in the final baked products, consistent with its effect on the dough.

Color measurements revealed that the lightness (L*) and yellowness (b*) values were the highest in the control group (27.63 and 12.10, respectively) and the lowest in the CP15 sample (21.80 and 5.43, respectively). Across the experimental groups, L* and b* values ranged from 21.80 to 27.23 and from 5.43 to 12.00, respectively, showing a significant decreasing trend with higher levels of cricket powder. In contrast, the redness value (a*) increased significantly, from 7.87 in the control to 11.67 in CP15, indicating that the addition of GB powder enhanced the red hue of the cookies. These changes in color may be attributed to the thermal denaturation of insect-derived proteins during baking [47]. Cookie color is primarily influenced by the Maillard reaction and caramelization, both of which occur at elevated temperatures in the oven and result in the formation of brown pigments on the surface [37].

In particular, melanoidins—brown, high-molecular-weight compounds formed during the Maillard reaction—may contribute to the observed increase in redness. Additionally, GB powder has a naturally darker hue compared to wheat flour, suggesting that the inherent color of the raw material also influenced the reduction in lightness and enhancement in redness in the final products. The lowest L* value observed in CP15 may be attributed to intensified Maillard and caramelization reactions driven by the higher concentration of insect powder. According to Martins et al. [48], when the pH of the dough is below 7, hexoses may undergo enolization followed by the formation of hydroxymethylfurfural (HMF), which can react with amino groups to produce brown-colored complexes. In the present study, the pH values of both control and experimental samples were below 7, suggesting favorable conditions for the formation of such brown pigments, thus amplifying the color changes. Interestingly, consumers often associate darker-colored baked goods with whole grain or health-oriented products. Therefore, the darker appearance of cookies observed in this study may potentially result in a positive perception among consumers [10].

### 3.4. Physical Properties of Cookies

The spread ratio and loss rate of chocolate cookies formulated with varying concentrations of GB powder are presented in Table 5. The highest spread ratio was observed in CP3 (12.68), while CP15 exhibited the lowest value (12.11). The control had a spread ratio of 12.46, which was lower than that of CP3. A significant decreasing trend in the spread ratio was observed as the level of insect powder increased from CP3 to CP15.

The spread ratio is a key quality indicator that reflects the extent to which cookie dough thins and spreads during baking and is influenced by multiple factors including dough viscosity, moisture content, and the levels of protein and dietary fiber [49]. Conventional commercial cookies are typically made from wheat flour-based dough, which forms a continuous gluten network during thermal processing and supports structural expansion until flow is arrested [44]. In contrast, the present study employed wheat starch to develop a gluten-free formulation and investigated the effects of partially substituting it with GB powder on the dough’s physical properties. At lower concentrations (e.g., CP3), the addition of cricket powder did not significantly increase dough viscosity, allowing the dough to spread more freely under the influence of gravity. However, at higher concentrations (≥9%), the increased protein and fiber content likely contributed to greater dough viscosity, thereby significantly reducing spreadability.

Proteins in the dough may enhance viscosity and, upon heating, undergo denaturation and aggregation, reinforcing the dough matrix and limiting flow. Furthermore, cricket powder contains a substantial amount of dietary fiber and chitin, which may enhance water absorption and retention capacity. This could reduce the availability of free water in the dough, influence sugar solubility, and increase dough dryness, ultimately contributing to reduced spread [50].

The loss rate was the lowest in CP15 (0.19) referring to the reduction in dough weight due to moisture and volatile compound evaporation during baking. A lower baking loss indicates better moisture retention, which contributes to a softer texture in the final product [31]. Previous research by Karim et al. [51] reported that high dietary fiber content increases the amount of bound water, thereby reducing moisture loss during thermal processing. In the present study, the incorporation of GB powder may have increased the fiber content of the dough, improving its water-holding capacity and contributing to lower baking loss.

### 3.5. Textural Analysis of Cookies

With the increasing prevalence of gluten-related disorders and increased interest in health-conscious diets, the demand for GF products has been growing. However, most GF products rely heavily on carbohydrate-based ingredients, resulting in nutritional limitations such as low levels of protein, dietary fiber, and essential minerals. To address these nutritional imbalances, this study utilized GB powder as a high-protein food ingredient. In addition, 3D printing technology was introduced to reduce consumers’ visual aversion to insect-based foods. Three-dimensional printing enables precise control over the shape and texture of food, making it suitable for the development of personalized functional products, especially for consumers with dietary restrictions. Accordingly, cookies were produced using 3D printing and post-processed to completion, after which their textural characteristics were evaluated. The effects of different concentrations of GB powder on the mechanical properties of the cookies are presented in Table 6. A significant increase in hardness was observed with increasing levels of cricket powder. Similarly, the total area under the force–time curve followed the same trend, with the control showing the lowest value and CP15 exhibiting the highest value. These increases in both hardness and total area may be attributed to changes in the physical characteristics of the dough induced by the addition of cricket powder. GB powder is rich in protein and dietary fiber, which likely contributed to an increase in dough viscosity and the formation of a stronger matrix during thermal processing through protein denaturation and network development. The presence of fiber may have also enhanced water absorption and retention, thereby increasing dough density and contributing to greater structural strength in the final baked product [51].

According to Culetu et al. [52], doughs with higher saturated fatty acid content tend to exhibit greater hardness, suggesting that the saturated fatty acids present in GB powder may have further contributed to the mechanical strength of the cookies. These findings are consistent with those reported by Bawa et al. [53], in which the incorporation of house cricket powder significantly increased the hardness and chewiness of both bread and cookies. This indicates that the protein and fiber components of insect powders can alter the rheological behavior of doughs, enhancing the structural integrity and textural attributes of the final product. In conclusion, the incorporation of GB powder significantly increased cookie hardness by elevating the protein and fiber content, thereby enhancing dough viscosity and structural cohesion.

These results suggest that insect powder has potential as a functional ingredient for modifying or improving the textural quality of cookies and may serve as a useful reference for future product development involving edible insects.

### 3.6. Consumer Test of Cookies

#### 3.6.1. Consumers’ Liking and RATA

The results of the consumer liking test for chocolate cookies containing varying concentrations of GB powder are presented in Table 7. The control group received an average acceptability score of 5.9, while CP3 obtained a slightly higher score of 6.1; however, the difference between the two was not statistically significant. A significant decreasing trend in acceptability was observed with increasing concentrations of cricket powder, indicating a dose-dependent negative effect. These findings are consistent with those of Draszanowska et al. [54], who reported a decline in overall liking as the level of mealworm powder in cookies increased. The results suggest that the distinctive flavor and coarse texture imparted by insect powder may adversely affect consumer acceptability, particularly at higher inclusion levels. High concentrations of insect powder may intensify unfavorable sensory attributes, leading to consumer rejection.

**Figure 3:**
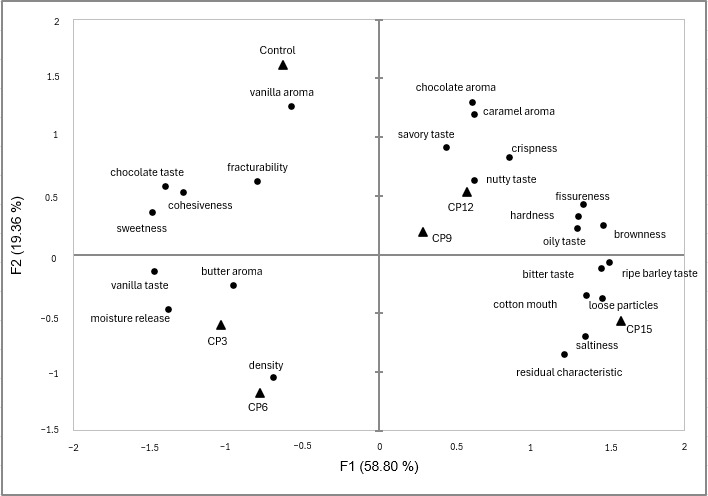
PCA plot using 24 RATA sensory attributes for cookies with different concentrations of *Gryllus bimaculatus* (GB) powder added. Product codes are same as those in Table 1.

**Figure 4:**
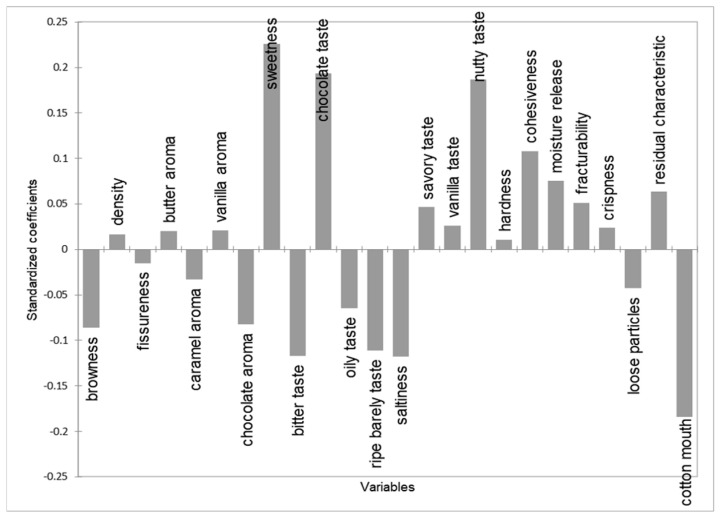
Plot of overall liking-standardized coefficients of PLSR of cookies with different concentrations of GB powder added.

PCA and PLSR were conducted to evaluate the sensory profiles and consumer responses to the cookies (Figure 3 and Figure 4). PCA was conducted using RATA data to comprehensively assess and visualize the dominant sensory attributes of each sample. This multivariate method projects high-dimensional sensory data into a lower-dimensional space, enabling effective differentiation among samples [55]. In the present study, PCA accounted for 78.18% of the total variance (F1: 58.80%; F2: 19.38%), allowing for clear separation based on sensory characteristics. The control was associated with attributes such as chocolate flavor, sweetness, and crumbliness—positive traits that align with the typical sensory expectations of chocolate cookies. CP3 and CP6 were characterized by a buttery aroma, vanilla flavor, and moist texture, suggesting that low levels of GB powder may enhance certain desirable sensory qualities. Conversely, CP9 and CP12 were associated with nutty and greasy notes, likely due to the presence of glutamic acid and aspartic acid—umami-enhancing amino acids naturally found in GB [41]. CP15 was linked to bitterness, astringency, and lingering aftertaste, all of which are considered negative attributes that may have contributed to the reduced acceptability. These findings support previous reports that certain amino acids in cricket powder, such as arginine, can produce bitter off-flavors [41,56].

PLSR was conducted to quantitatively determine the influence of sensory attributes on overall acceptability (Figure 2). The model enabled the visualization of the relationship between sensory descriptors and consumer liking responses. In this analysis, sensory variables with a variable importance in projection (VIP) score ≥ 1.0 were considered to have a significant impact on consumer preference [57]. The key drivers identified were sweetness, chocolate flavor, moistness, nuttiness, saltiness, bitterness, astringency, and roasted barley flavor. According to the standardized coefficient results (Figure 2), sweetness, chocolate flavor, moistness, and nuttiness positively influenced acceptability, whereas bitterness, astringency, roasted barley flavor, and saltiness had negative effects. These findings are consistent with the observation that CP15 received the lowest acceptability score and suggest that bitterness and astringency, intensified by higher concentrations of GB powder, are critical factors contributing to consumer rejection. Overall, the sensory modifications resulting from the incorporation of cricket powder had a direct impact on consumer acceptability. These results provide important insights for determining the optimal level of GB addition in order to maintain desirable sensory qualities and consumer acceptance in insect-based cookie formulations.

#### 3.6.2. Survival Analysis

**Figure 5:**
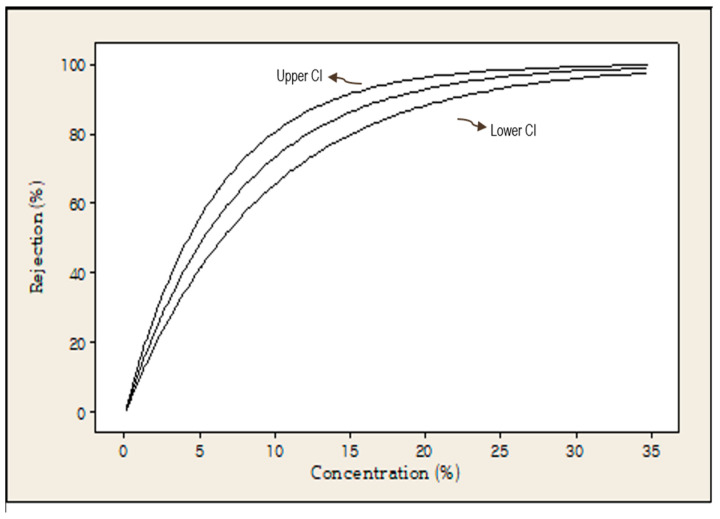
Probability of consumer rejecting cookies compared to GB powder concentration for exponential distribution (Mid line: predicted probability; CI: 95% confidence interval).

Although CP3 exhibited the highest acceptability score among the samples, identifying it as the optimal concentration of GB powder may result in limited precision due to individual variation in consumer preference and perception. It is possible that similar levels of acceptability may be observed in adjacent concentration ranges such as 0–3% or 3–6%. To reduce this uncertainty and determine a more precise threshold for consumer acceptability, survival analysis was conducted. The results are summarized in Table 8 and Table 9 and Figure 5.

Table 8 presents a summary of consumer response patterns based on the acceptance or rejection of samples, along with the corresponding censoring classifications. Subject 1 accepted samples from 0 to 9% but rejected the 12% sample; however, the exact rejection threshold was indeterminable, and thus this case was categorized as interval-censored. A total of 54 participants displayed this response pattern. Subject 2 rejected all concentrations, suggesting a personal threshold below the lowest level presented (0%), and was therefore classified as left-censored. This response type was observed in 29 participants. Subject 3 displayed an inconsistent response—rejecting the 6% sample but accepting the 9 and 12% samples and again rejecting 15%. In this study, such irregular cases were assumed to have a threshold between 3 and 6% and were treated as interval-censored, with 22 participants falling into this category. Subject 4 accepted all concentrations up to 15%, indicating a threshold above the maximum tested level, and was classified as right-censored (n = 11). Subject 5 rejected the control sample but accepted the subsequent samples, showing inconsistent response behavior that precluded meaningful threshold estimation; this group (n = 6) was excluded from further analysis.

Figure 5 presents the rejection probability curve derived from the exponential distribution model, including a 95% confidence interval. The analysis estimated that the GB powder concentration at which 50% of consumers were expected to reject the cookie was 5.23%. In survival analysis for sensory evaluation, rejection thresholds at which 25 or 50% of consumers reject a product are commonly used to define consumer tolerance limits. This provides quantitative indicators for determining sensory quality degradation based on actual consumer perception [28]. In this context, the estimated 50% rejection point represents the concentration at which half of the population is likely to either accept or reject the product. Unlike arbitrarily defined sensory cutoffs, consumer-based survival analysis offers a more objective and reliable method for defining quality limits.

Table 9 summarizes the estimated parameters for GB powder concentration based on the exponential distribution model, including the mean, standard error, median (50% rejection), and the concentrations corresponding to the 25 and 75% rejection probabilities. The average rejection concentration was estimated at 7.54%, indicating the mean level at which consumers are likely to reject the product. The estimated concentration at which 25% of consumers rejected the sample was 2.17%, and the concentration at which 75% rejected it was 10.45%. These results demonstrate a clear trend: as the concentration of GB powder increases, the probability of consumer rejection also increases. This confirms again that the sensory characteristics associated with higher levels of insect powder may have a negative impact on overall acceptability.

### 3.7. Correlation Analysis

The correlation analysis between overall acceptability and physicochemical characteristics (Table 10) provided meaningful insights into how the addition of insect powder affects the sensory acceptability of gluten-free cookies. Notably, color parameters such as L* and b* showed positive correlations with overall acceptability (r = 0.87, *p* < 0.05), suggesting that a bright and vivid appearance positively influenced consumer preferences. In contrast, a* exhibited a strong negative correlation with acceptability (r = −0.93, *p* < 0.05), indicating that excessive browning or darker hues caused by increased insect powder concentration may negatively impact consumer perception.

Crude ash and crude fat contents also showed strong negative correlations with overall acceptability (r = −0.90, *p* < 0.05). These results imply that elevated levels of minerals and fat—resulting from the addition of insect powder—may have adversely affected the flavor and texture of the product. In particular, higher ash content may be associated with off-flavors or a chalky mouthfeel attributed to the intrinsic properties of the insect material, while increased fat content could have contributed to lipid oxidation or excessive greasiness, thereby compromising sensory quality. These findings suggest that compositional properties not only contribute to the nutritional benefits of insect powder but also influence sensory perception.

Furthermore, both cookie and dough pH exhibited strong positive correlations with overall acceptability (r = 0.90, *p* < 0.05), indicating that pH levels may play a role in enhancing taste, aroma, and texture. The pH shifts induced by insect powder incorporation could therefore be considered a critical determinant of consumer acceptance. Taken together, these results confirm that the addition of insect powder induces significant changes in the physicochemical properties of cookies, which in turn directly influence consumer acceptability.

## 4. Conclusions

This research contributes quantitative insights into consumer rejection limits for insect-based ingredients, providing a direction for the future development of edible insect products. The key contribution of this study lies in its comprehensive evaluation of the quality characteristics and consumer acceptability of GF cookies enriched with edible insect powder and the application of survival analysis to quantitatively determine rejection probabilities. However, it should be noted that the cookies in this study were produced using a 3D food printing process, which presents inherent challenges due to the sensitivity of dough rheology to process parameters. Variables such as dough viscosity, extrusion speed, and nozzle diameter can significantly affect the structural and textural attributes of printed products. Particularly, insect powders such as GB powder may respond sensitively to these changes, potentially compromising product consistency in commercial-scale applications. In addition, the incorporation of insect powder may alter lipid and protein fractions, potentially reducing oxidative stability and increasing susceptibility to microbial growth during storage and raising concerns about the possible presence of chemical contaminants such as heavy metals and residues. These factors should be acknowledged as limitations of the present study. Therefore, future research should focus on optimizing key 3D printing parameters—such as printing speed, nozzle diameter, and extrusion pressure—to mitigate the variations in dough behavior caused by GB addition. To achieve such optimization, it would be advisable to employ experimental design approaches such as central composite rotatable design (CCRD) and simplex-centroid design. Moreover, further studies are needed to evaluate the quality, microbiological characteristics, potential chemical contaminants (e.g., heavy metals and residues), and consumer acceptance of insect-based foods across diverse food matrices and processing techniques. Such research will be essential in enhancing the commercial viability of edible insect products and promoting their use as sustainable alternative protein sources.

## Figures and Tables

**Table 1:** Formula of gluten-free (GF) chocolate cookies added with different concentrations of *Gryllus bimaculatus* (GB) powder.

Materials	Groups (g/100 g) ^1^					
	Control	CP3	CP6	CP9	CP12	CP15
Wheat starch	20	17	14	11	8	5
Cricket powder	0	3	6	9	12	15
Refined sugar	25	25	25	25	25	25
Unsalted butter	17	17	17	17	17	17
Eggs	20	20	20	20	20	20
Cocoa powder	15	15	15	15	15	15
Vanilla extract	2	2	2	2	2	2
Salt	1	1	1	1	1	1
Total	100	100	100	100	100	100

^1^ Control: cookie prepared without GB powder; CP3: cookie prepared with 3 g of GB powder; CP6: cookie prepared with 6 g of GB powder; CP9: cookie prepared with 9 g of GB powder; CP12: cookie prepared with 12 g of GB powder; C15: cookie prepared with 15 g of GB powder.

**Table 2:** The proximate composition of GF chocolate cookies with different concentrations of GB powder added. (Unit: %.)

Groups	Moisture	Crude Protein	Crude Fat	Crude Ash
Control	4.18 ± 0.04 ^a,1,2^	15.00 ± 0.08 ^a^	15.84 ± 0.50 ^e^	1.60 ± 0.01 ^f^
CP3	4.20 ± 0.01 ^a^	26.28 ± 0.09 ^b^	17.16 ± 0.61 ^d^	1.69 ± 0.01 ^e^
CP6	4.21 ± 0.05 ^a^	28.98 ± 0.06 ^c^	20.83 ± 0.56 ^c^	1.77 ± 0.00 ^d^
CP9	4.21 ± 0.03 ^a^	31.10 ± 0.04 ^d^	22.35 ± 0.51 ^b^	1.84 ± 0.00 ^c^
CP12	4.22 ± 0.05 ^a^	32.78 ± 0.04 ^e^	23.28 ± 0.10 ^a^	1.95 ± 0.02 ^b^
CP15	4.26 ± 0.01 ^a^	36.31 ± 0.06 ^f^	24.20 ± 0.6 ^a^	2.05 ± 0.01 ^a^

Product codes are same as those in Table 1. ^1^ Mean values are mean ± SD (n = 3). ^2^ Means with different letters (a–f) in same column are significantly different at *p* < 0.05 according to Tukey’s HSD test.

**Table 3:** pH and density of GF chocolate cookie dough with different concentrations of GB powder added.

Groups	pH	Density (g/mL)
Control	7.24 ± 0.02 ^a,1,2^	1.23 ± 0.07 ^a^
CP3	6.86 ± 0.01 ^b^	1.24 ± 0.01 ^a^
CP6	6.60 ± 0.01 ^c^	1.21 ± 0.08 ^a^
CP9	6.43 ± 0.01 ^d^	1.20 ± 0.06 ^a^
CP12	6.35 ± 0.01 ^e^	1.24 ± 0.05 ^a^
CP15	6.16 ± 0.01 ^f^	1.21 ± 0.09 ^a^

Product codes are same as those in Table 1. ^1^ Mean values are mean ± SD (n = 5). ^2^ Means with different letters (a–f) in same column are significantly different at *p* < 0.05 according to Tukey’s HSD test.

**Table 4:** pH and color values of cookies with different concentrations of GB powder added.

Groups	pH	L*	a*	b*
Control	6.97 ± 0.01 ^a,1,2^	27.63 ± 0.46 ^a^	7.87 ± 0.12 ^e^	12.10 ± 0.40 ^a^
CP3	6.79 ± 0.00 ^b^	27.23 ± 0.21 ^ab^	9.03 ± 0.12 ^d^	12.00 ± 0.17 ^a^
CP6	6.75 ± 0.01 ^c^	26.97 ± 0.23 ^b^	9.30 ± 0.10 ^d^	9.77 ± 0.35 ^b^
CP9	6.62 ± 0.02 ^d^	26.03 ± 0.42 ^c^	10.30 ± 0.53 ^c^	6.83 ± 0.59 ^c^
CP12	6.52 ± 0.01 ^e^	22.87 ± 0.71 ^d^	11.00 ± 0.17 ^b^	7.23 ± 0.40 ^c^
CP15	6.44 ± 0.02 ^f^	21.80 ± 0.52 ^e^	11.67 ± 0.15 ^a^	5.43 ± 0.12 ^d^

Product codes are same as those in Table 1. ^1^ Mean values are mean ± SD (n = 5). ^2^ Means with different letters (a–f) in same column are significantly different at *p* < 0.05 according to Tukey’s HSD test.

**Table 5:** Spread ratios and loss rates of cookies with different concentrations of GB powder added.

Groups	Spread Ratio	Loss Rate (%)
Control	12.46 ± 0.12 ^b,c,1,2^	0.20 ± 0.01 ^b^
CP3	12.68 ± 0.16 ^a^	0.21 ± 0.01 ^a^
CP6	12.51 ± 0.39 ^ab^	0.20 ± 0.00 ^b^
CP9	12.44 ± 0.26 ^bc^	0.20 ± 0.00 ^b^
CP12	12.27 ± 0.25 ^bc^	0.20 ± 0.00 ^bc^
CP15	12.11 ± 0.24 ^c^	0.19 ± 0.00 ^c^

Product codes are same as those in Table 1. ^1^ Mean values are mean ± SD (n = 5). ^2^ Means with different letters (a–c) in same column are significantly different at *p* < 0.05 according to Tukey’s HSD test.

**Table 6:** Textural parameters of cookies with different concentrations of GB powder added.

Groups	Peak (g)	Total Area (g × s)
Control	2812.30 ± 558.20 ^b,1^	4569.5 ± 777.81 ^b^
CP3	2876.65 ± 332.82 ^ab^	4881.07 ± 618.92 ^ab^
CP6	3140.17 ± 522.10 ^ab^	4882.80 ± 412.34 ^ab^
CP9	3145.34 ± 696.13 ^ab^	4890.85 ± 549.01 ^ab^
CP12	3181.65 ± 477.54 ^ab^	5241.38 ± 486.31 ^a^
CP15	3245.73 ± 468.57 ^a^	5332.18 ± 715.45 ^a^

Product codes are same as those in Table 1. ^1^ Means with different letters (a,b) in same column are significantly different at *p* < 0.05 according to Tukey’s HSD test.

**Table 7:** Overall liking scores of cookies with different concentrations of GB powder added in consumer preference test.

Groups	Overall Liking
Control	5.9 ± 1.5 ^a,1,2^
CP3	6.1 ± 1.5 ^a^
CP6	5.4 ± 1.4 ^b^
CP9	4.8 ± 1.5 ^c^
CP12	4.6 ± 1.4 ^c^
CP15	4.0 ± 1.5 ^d^

Product codes are same as those in Table 1. ^1^ Mean values are mean ± SD (n = 100). ^2^ Means with different letters (a–d) in same column are significantly different at *p* < 0.05 according to Tukey’s HSD test.

**Table 8:** Acceptance and rejection results for five typical panelists who tasted cookies with different concentrations of GB powder added.

Panel	Concentration (%)						Censoring
	0	3	6	9	12	15	
1	yes	yes	yes	yes	no	no	Interval: 9–12
2	no	no	no	no	no	no	Left: 0
3	yes	yes	no	no	yes	no	Interval: 3–6
4	yes	yes	yes	yes	yes	yes	Right: 15
5	no	yes	no	no	yes	no	Not considered

**Table 9:** Parameters for GB powder concentration of cookies estimated using exponential distribution. (Unit: %.)

Parameter	Estimation	Standard Error	Lower CI ^1^	Upper CI
Mean	7.54	0.83	6.08	9.36
Standard deviation	7.54	0.83	6.08	9.36
Median	5.23	0.58	4.21	6.48
25% rejection	2.17	0.24	1.75	2.69
75% rejection	10.45	1.15	8.42	12.97

^1^ CI, 95% confidence interval.

**Table 10:** Pearson’s correlation coefficients between consumer acceptability and physicochemical parameters of 3D-printed GF cookies with GB powder.

	Spread Ratio	L*	OL	b*	Crude Ash	Total Area	Cookie pH	a*	Crude Fat	Peak	Dough pH	Crude Protein
Spread ratio		0.896 *	0.946 **	0.885 *	−0.860 *	−0.743	0.799	−0.825 *	−0.814 *	−0.774	0.761	−0.626
L*			0.869 *	0.851 *	−0.963 **	−0.939 **	0.921 **	−0.929 **	−0.835 *	−0.765	0.807	−0.772
OL				0.975 ***	−0.893 *	−0.797	0.898 *	−0.916 *	−0.922 **	−0.890 *	0.908 *	−0.800
b*					−0.885 *	−0.811	0.936 **	−0.946 **	−0.966 **	−0.923 **	0.953 **	−0.855 *
Crude ash						0.976 ***	−0.948 **	0.966 **	0.919 **	0.886 *	−0.907 *	0.891 *
Total area							−0.946 **	0.952 **	0.865 *	0.819 *	−0.872 *	0.908 *
Cookie pH								−0.997 ***	−0.937 **	−0.872 *	0.947 **	−0.933 **
a*									0.953 **	0.899 *	−0.959 **	0.938 **
Crude fat										0.984 ***	−0.987 ***	0.926 **
Peak											−0.971 **	0.912 *
Dough pH												−0.966 **
Crude protein												

*p* < 0.05 *, *p* < 0.01 **, *p* < 0.001 ***; values are statistically significant. OL = overall liking.

## Data Availability

The data presented in this study are available on request from the corresponding author. The data are not publicly available due to privacy protection and ethical restrictions.

## Abbreviations

GB	Gryllus bimaculatus
GF	Gluten-free
PLSR	Partial least squares regression
PCA	Principal component analysis
